# Mothers and Fathers Parenting Stress and Their Perception of Children’s Psychosocial Functioning in Paediatric Diabetes: A Pilot Study

**DOI:** 10.3390/ijerph17134734

**Published:** 2020-07-01

**Authors:** Daniela Di Riso, Giulia Bassi, Elisa Mancinelli, Silvana Zaffani, Silvia Salcuni, Claudio Maffeis

**Affiliations:** 1 Department of Developmental Psychology and Socialization, University of Padova, 35122 Padova, Italy; daniela.diriso@unipd.it (D.D.R.); silvia.salcuni@unipd.it (S.S.); 2 Fondazione Bruno Kessler, 38123 Trento, Italy; 3 Department of General Psychology, University of Padova, 35131 Padova, Italy; elisa.mancinelli@studenti.unipd.it; 4 Pediatric Diabetes and Metabolic Disorders Unit, Department of Surgery, Dentistry, Pediatrics and Gynaecology, University-Hospital of Verona, 37126 Verona, Italy; silvana.zaffani@aovr.veneto.it (S.Z.); claudio.maffeis@univr.it (C.M.)

**Keywords:** parent couples, pediatric diabetes, type 1 diabetes mellitus, parental perception, parenting stress

## Abstract

(1) **Background**: In the context of a child with Type 1 Diabetes Mellitus (T1DM), the rearrangement of the family’s lifestyle can account for an increased risk of experiencing psychosocial problems for both child and parents. Those few studies on pediatric diabetes, which focused on parents’ perception of children’s psychological strengths and weaknesses, reported significantly higher rates of children’s emotional and conduct problems associated with an imbalance in the Hemoglobin A1c (HbA1c). The main aim of this paper was to assess the role of parental perception of children’s psychosocial symptoms as a mediator of the perceived parenting stress, considering mother and father separately. (2) **Methods**: The study involved 12 parent couples (Mothers M_age_ = 40.25, SD = 6.58; Fathers M_age_ = 42.5, SD = 6.38) of children with T1DM aged between 7 and 11 years (M_age_ = 8.8, SD = 0.996). Parents completed questionnaires such as the Strengths and Difficulties Questionnaire for parents and their perspective of their child, and the Parenting Stress Index–Short Form. (3) **Results**: Mothers and fathers had significant differences in the perception of their child’s internalizing symptoms. Specifically, mothers present a greater perception of the mentioned symptoms compared to fathers. Mediation models showed that only for fathers’ perception of the child conduct problems has a significant role between the fathers’ perception of dysfunctional interaction with the child and the HbA1c. (4) **Conclusions**: The current study provides useful evidence also for clinical settings, suggesting that an interesting interplay between parenting stress, perception of children’s symptoms and glucometabolic control should be taken into consideration.

## 1. Introduction

Type 1 Diabetes Mellitus (T1DM) is one of the most prevalent chronic diseases with onset in childhood worldwide [1]. Recent epidemiological data indicates that about 20,000 Italian children and adolescents have been diagnosed with T1DM, with incidence rates increasing with age, from birth to 17 years old (3%) [2]. Although in Italy most of the T1DM onset usually occurs when children are between 9 and 11 years old, annual incidence rates are most rapidly increasing in preschool-aged children (6%) [2,3]. T1DM entails specific self-care practices that can become invasive during one’s everyday life, such as happens with constant blood glucose monitoring and insulin administration, often associated with rigorous diet regimens, sports activities and hospitalization periods. In families with a child with T1DM, the associated internal re-organization of family roles and dynamics, together with the uncertainty caused by possible acute complications [4], might account for the increased risk of experiencing psychosocial problems on the part of both children and parents [3,5,6]. Authors suggest that, where T1DM is concerned, the psychological functioning of parents and children are mutually related, thereby directly influencing each other [7]. In the specific case of parenting a child with T1DM, parenting stress is influenced by factors such as the responsibility felt toward the child’s diabetes-management and anxious states concerning the fear of hypoglycemia [8,9,10,11] as well as the frequency of hypoglycemia, the number of years since diagnosis and demographic variables such as marital status and education level [12]. Indeed, parents’ diabetes-specific stress can have repercussions on their well-being [4], and in turn on that of their children, thereby potentially influencing the child’s Hemoglobin A1c (HbA1c), and also on their depression symptoms [13,14,15]. As such, one could speculate on how the psychological and behavioral adjustment of the child to the disease, as well as to its glycemic values, as critical components intrinsic to T1DM, influence the psychological functioning of parents; especially since parenting stress seems to be one of the core factors leading to increasing psychosocial difficulties within families of children with T1DM [16,17]. Moreover, perception of the child’s well-being is impacted in parents facing paediatric T1DM, compared to families without chronic illnesses, and affects the mother’s behavior and psychological well-being differently from the father’s [18]. For example, Van Gampelaere and colleagues (2019) reported that mothers show the greater perception of difficulties in the child’s adjustment to the disease as compared to fathers. Such differences were accounted for by the higher maternal stress reported, based also on the assumption that the mothers are more receptive to the child’s behavioral problems [18]. In this context, parenting stress referred to parenting a child with T1DM is indeed shown to be influenced by their rating of the child’s emotional and behavioral problems [17]. Studies investigating the child’s and the parent’s psychosocial factors relevant within T1DM have mainly considered mothers, while investigating neither the role of fathers nor their experiences, in any particular depth [19,20]. Despite this, the literature stressed the importance of taking into account both mother and father, in a multi-informant approach, to assess mental health in children [21]. Indeed, within pediatric T1DM, most studies have only included mothers in their sample [20,21,22,23,24,25], often evaluating mother–child reciprocal depressive symptoms [13,26], how mothers’ parenting influences the child’s glycemic control [25] or reporting an association between maternal parenting stress and the child’s internalizing and externalizing problems [27]. Conversely, studies that considered fathers did not analyze them and their psychological function related to the management of a child with T1DM so specifically, most often due to the uneven presence of both mothers and fathers within the samples [28]. Few studies took fathers into account as mothers were considered the primary caregivers involved in the management of the chronic illness of their children, such as insulin administration and the following of a specific diet [23,29,30]. On the other hand, among studies taking the father’s psychosocial variables into account, these were most often related to anxiety, depression, and parenting stress [4,8,31].

Findings show that a father’s perception of greater involvement in diabetes-management is associated with increased anxiety and parenting stress, thereby resulting in the child’s poorer glycemic control. However, paternal investment in the child’s diabetes-care as perceived by mothers, but not the effective paternal participation, associates with better marital satisfaction and HbA1c levels in their child [32]. On the other hand, fathers with a positive self-perception show greater involvement in their child’s diabetes-management, resulting in improved adherence to the child’s illness regimen, further associating with better HbA1c levels [8,31]. The differences between the above findings might be explained by the perception of the child’s psychosocial variables. Paternal paediatric parenting stress appears to be less frequent compared to that experienced by mothers [32].

Furthermore, a fathers’ pediatric parental stress is related to state anxiety and the mother’s stress reports of impaired child psychosocial functioning [31]. Few studies found relationships between paternal stress and the child’s psychosocial factors. Authors indicate that a child’s mood, as well as their distractibility, are central in the increase in paternal stress, a pattern that seems to be associated with higher levels of reported fathers’ parenting stress when caring for a child with diabetes [31]. As such, it could be expected that fathers could differ from mothers when experiencing parenting stress together, with several implications in the management of the child’s diabetes [31,32]. A few studies regarding paediatric T1DM have instead investigated parents’ perception of their child’s psychological state as related to the child’s strengths and weaknesses. Findings highlighted that parents of children with T1DM report significantly more child psychosocial symptoms, as measured through the Strengths and Difficulties Questionnaire–Parent version, compared to parents of medically healthy children. In particular, a child’s emotional and behavioral problems are perceived as worse by parents of children with T1DM [3,33,34]. Two studies also reported a moderately significant correlation between parents’ reports of their child’s emotional, behavioral problems and a scarce control of their child’s HbA1c levels [35,36]. Moreover, studies show that the more parents report a child’s psychosocial impairment, the more they present difficulties associates with parenting stress [30,34] and poorer glycemic control in the child’s diabetes [36]. Still, the question remains as to how parenting stress within pediatric T1DM is influenced by the child’s HbA1c levels [13,14,15].

As such, the general aim of the present paper is to analyze the differences between the maternal and paternal perception of the child with T1DM and their parenting stress. More specifically, the purpose is to assess the role of parents’ perception of children’s psychosocial symptoms as a mediator between perceived parenting stress and children’s HbA1c levels.

Relying on past literature, it has been hypothesized that:

1. mothers and fathers would differ in respect to the levels of parenting stress and perception of their child’s psychosocial symptoms [18] as they are often reported to be involved differently in the intense diabetes-management regimen [23,29,30];
2. the child’s HbA1c would significantly correlate with both mothers’ and fathers’ parenting stress and their perception of the child’s psychosocial symptoms [6];
3. a relationship between HbA1c and mothers’ and fathers’ parenting stress, mediated by perception of their child’s emotional and behavioral problems. It was assumed that poor glycemic control would be associated with a higher level of parenting stress, mediated by their association with the increased perception of the child’s emotional and behavioral problems in both parents [8,9,10,11,13].

## 2. Materials and Methods

### 2.1. Participants

The present study initially involved 47 parents (*n* = 29 mothers; N = 18 fathers) of 33 children diagnosed with T1DM. However, upon request for participation in the study to the spouses of 23 parents (*n* = 17 mothers; *n* = 6 fathers), 18 refused to participate. The study was thus composed of 18 parental couples. However, 5 parents did not fully complete the measures, resulting in a sample of 12 parental couples of 12 children (8, 66.7% females) diagnosed with T1DM, at least 1 year prior to study enrolment, confirmed by positivity of at least one of the antibodies against islet cells (ICA), insulin (IAA), glutamate dehydroxilase (GADA), islet antigen 2 (IA2A) and zinc-transporter protein 8 (ZnT8A). Parents’ age ranged between 28–50 years (Mothers Mage = 40.25, SD = 6.58; Fathers Mage = 42.5, SD = 6.38). Of the included parents, 36.4% of mothers and 36.4% of fathers were middle school graduated, 36.4% of mothers and 54.5% of fathers were high school graduated, while 27.3% of mothers and 9.1% of fathers had a university degree. At the time of data collection, children’s age ranged between 7 and 10 years (Mage = 8.92, SD = 0.996) and they were diagnosed with insulin-dependent T1DM from at least 24 months (M = 47.00, SD = 21.38). Participants included in the analysis satisfied the following inclusion criteria, as (i) participation was limited to parental couples of children diagnosed with T1DM (ii) with Hb1Ac equal or higher than 42 mmol/mol (6%). In the current paper, children present a HbA1c mean level of 57 mmol/mol (7.3%, SD = 10.427). 

### 2.2. Procedure 

Sample recruitment and questionnaire administration were conducted in the Paediatric Diabetes and Metabolic Disorders Unit, of the University Hospital in Verona (Italy) by trained psychology master students with the supervision of the ward’s psychotherapist. The research project was presented to parents who accompanied their children to a scheduled medical visit. Mothers and fathers completed measures in the Hospital upon signed and oral consent. The recruitment period was set at 2 months according to the Hospital ward and Ethical Committee. Children’s medical information was retrieved from medical charts. All subjects were informed of the confidentiality of data and that they could withdraw from the study at any moment. The study procedure followed the Declaration of Helsinki (Italian law 196/2003, UE GDPR 679/2016) and was approved by the Hospital Ethical Committee (code: VADMT1CGM2018).

### 2.3. Measures

#### 2.3.1. The Strengths and Difficulties Questionnaire–Parents

The Strengths and Difficulties Questionnaire–Parents (SDQ-P) [37] is a screening measure used in clinical and research practice. It is a brief questionnaire comprising 25 items measured on a 3-point Likert scale (1 = “Not True”; 2 = “Somewhat true”; 3 = “Certainly true”). It is aimed at assessing children’s and adolescents’ strengths and difficulties as perceived by their parents. The questionnaires have 5 subscales, namely *Emotional symptoms*, *Conduct problems*, *Hyperactivity-Inattention*, *Peer problems* and *Prosocial behaviour*, and a *Total Difficulty* score resulting from the sum of the first four subscales. SDQ-P scores also provide two other scales, namely *Internalizing problems* (as the sum of the sub-scales Emotional symptoms and Peer problems) and *Externalizing symptoms* (as the sum of the sub-scales Conduct problems and Hyperactivity-Inattention). Examples of items are as follows, *“Restless, overactive, cannot stay still for long”* (item 2), *“Many worries, often seems worried”* (item 8), and “*Picked on or bullied by other children”* (item 19). The instrument was also validated in Italy [38] on a sample of children and adolescents aged 6–13 years. Parents had to refer to the previous 6 months, or the year in which assessment took place, with higher scores indexing greater perceived difficulties and lower scores indexing greater perceived positive attributes of children. The SDQ-P Italian version [38] shows an internal validity of Cronbach’s α 0.81. In the current study, Cronbach’s α reliability coefficient of the SDQ 5 subscales were 0.95 for Emotional symptoms, 0.95 for Conduct problems, 0.66 for Hyperactivity-Inattention, 0.97 for Peer problems, 0.41 for Prosocial behaviour, 0.96 for Total Difficulty Score, 0.90 for Internalizing Symptoms and 0.87 for Externalizing Symptoms.

#### 2.3.2. Parenting Stress Index-Fourth Edition-Short Form 

The Parenting stress index-Fourth Edition–Short Form (PSI-4-SF) [39] is a brief screening tool rated on a 5-point Likert scale and comprising 36 items equally subdivided into three subscales, namely *Parental distress*, *Parent–child dysfunctional interaction* and *Difficult child*. The instrument further provides a *Total Stress scale* resulting from the sum of all subscales. The PSI-4-SF is aimed at identifying problem areas in parent–child interactions and clinically significant parenting stress as indexed by the Total Stress scale resulting from the parental-role. It also identifies aspects leading to potentially problematic behavior of the child or the parent. The instrument also comprehends a *Defensive response* scale evaluating distorted answers apt at providing a more favorable image of the parent. Sample items are as follows, *“Feel that I cannot handle things”* (item 1), *“Having a child caused problems with spouse”* (item 8), and *“Child is moody and easily upset”* (item 27). The Italian validation was performed on a sample of parents with children aged 0–10 years [40]. Furthermore, Cronbach’s α reliability coefficient of the PSI-4-SF 3 subscales were 0.82 for Parental Distress, 0.84 for Parent–child dysfunctional interaction, 0.90 for Difficult child and 0.62 for Total Stress scale.

### 2.4. Statistical Analysis

A Wilcoxon test for independent samples was performed to test differences between couples of parents in the parental perception of the positive and negative attributes of their child (assessed by SDQ-P) and parenting stress (assessed by PSI-4-SF). In this regard, the PSI-4-SF scale (i.e., Total Stress) and subscales (i.e., Parental Distress, Difficult Child, Parent–Child Dysfunctional Interaction) were evaluated. 

Pearson’s partial correlations (two-tailed) were carried out between couples of parents in the parental perception of the positive and negative attributes of their child, parenting stress and HbA1c (mmol/L, mg/dL) of the child, controlling for the time of diagnosis in months. Cohen’s d was used to evaluate the effect size for power analysis goals [41]. Following Cohen’s guidelines (1988), the effect size has been categorized as low (*d* = 0.2), medium (*d* = 0.5) and high (*d* = 0.8).

Two mediation models were performed for both mothers and fathers, using HbA1c (mg/L) of the child with T1DM as the independent variable, the subscales of the PSI-4-SF questionnaire (i.e., Parent–Child Dysfunctional Interaction) as the dependent variable and the SDQ-P two subscales (i.e., Emotional and Conduct Problems) as mediators. The bootstrapping method was applied to all the mediation models [42]. Specifically, a 1000 bootstrap sample was drowned from the full data and a 95% Confidence Interval (CI) was used to determine the significance of the mediating effect, and it was able to develop an empirical approximation of the sampling distribution of αβ by repeatedly sampling the dataset. More specifically, the bootstrapping procedure is a non-parametric inferential technique though which it is possible to create an empirical representation of the sampling distribution regarding the indirect effect by treating the obtained sample of size n as a representation of the population in miniature [43]. After that, during the analyses the sample is repeatedly resampled, thereby mimicking the original sampling procedure. Following the construction of a resample, a and b are estimated on such a dataset and the result of the path coefficients recorded [44]. Indeed, a recent study used a sample of 15 hypothetical caregivers in order to demonstrate the feasibility of the mediational model using the bootstrapping method with a 95% Confidence Interval, as reported by Levy and colleagues [44]. The significant mediating effect has been identified when the CI excluded zero and thus the null hypothesis should be rejected. 

Statistical analyses were carried out using IBM Corp SPSS Statistics, Version 24.0, Armonk, New York [45] and the package SPSS macro (Process, Model 4) [42]. A *p*-value < 0.05 was considered statistically significant.

## 3. Results

In the current study, all the variables show a normal distribution. 

### 3.1. T-Test For Paired-Samples in Parental Couples

No differences for PSI-4SF Total Stress and subscales were found (Table 1). According to SDQ-P, mothers had a significantly (*p* < 0.05) higher perception of child Internalizing Symptoms compared to fathers. No differences emerged for the other variables.

### 3.2. Partial Correlations in Parental Couples

As shown in Table 2, in the group of mothers, children’s HbA1c showed positive and significant correlations with PSI_Difficult Child, with PSI_Parent–Child Dysfunctional Interaction. Furthermore, child’s HbA1c had positive and significant (*p* < 0.05) correlations with SDQ-P_Internalizing and SDQ-P_Externalizing symptoms, and in particular with SDQ-P_Conduct Problems and SDQ-P_Emotional Problems. According to Cohen’s d, the aforementioned correlations presented a high effect size.

As reported in Table 3, findings for paternal variables indicated that children’s HbA1c significantly correlated with PSI_Difficult Child and SDQ-P_Conduct Problems, also increasing the overall levels of PSI_Total Stress and PSI_Parental Distress. Furthermore, in the group of fathers, HbA1c of the child significantly correlated with SDQ-P_Internalizing and SDQ-P_Externalizing symptoms, and in particular with SDQ-P_Conduct Problems and SDQ-P_Emotional Problems. According to Cohen’s *d*, the aforementioned correlations are characterized by a high effect size.

Therefore, the glycemic level of the child is positively and significantly associated with PSI_Difficult Child, with SDQ-P_Internalizing and with SDQ-P_Externalizing symptoms, in particular with SDQ-P_Conduct Problems and SDQ-P_Emotional problems, as shown in both parents.

### 3.3. Mediation Models

#### 3.3.1. Fathers

The relation between HbA1c) and SDQ-P_Conduct Problems was significant (F_(1,9)_ = 38.30, *p* < 0.001, R^2^ = 0.81). Moreover, the full mediation model was significant (F_(1,9)_ = 11.75, *p* = 0.004) accounting for 75% of the explained variability in the PSI_Parent–Child Dysfunctional Interaction.

**Figure 1:**
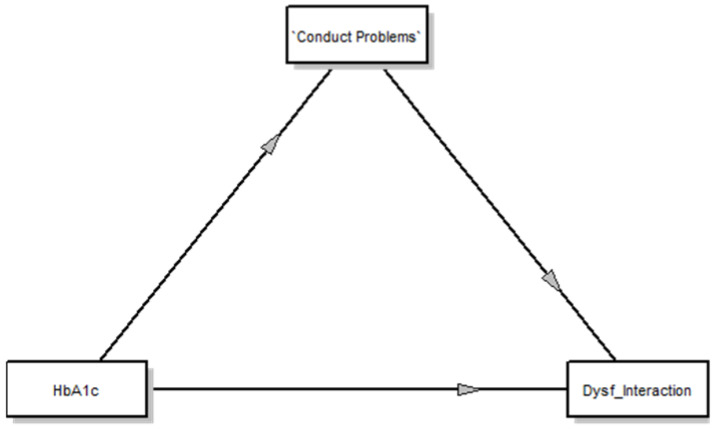
Mediation Model of Father Perception of their child with T1DM. Note. HbA1c = Hemoglobin A1c; Conduct Problems (assessed by SDQ-P); Dysf_Interaction = Parent–Child Dysfunctional Interaction (assessed by PSI-4-SF).

The total (non-mediated) model of HbA1c on PSI_Parent–Child Dysfunctional Interaction was significant (β = 0.09, t = 2.43, *p* = 0.04, 95% CI = 0.01, 0.17). HbA1c showed both direct (β = 0.25, *t* = 4.53, *p* < 0.001, 95% CI = 0.12, 0.38) and indirect effects on PSI_Parent–Child Dysfunctional Interaction (PSI-4-SF) as mediated through SDQ-P_Conduct Problems (β = −0.17, SE = 0.07, 95% CI = −0.29, −0.04). Therefore, SDQ-P_Conduct Problems was a significant mediator, as shown in Figure 1.

#### 3.3.2. Mothers

The model shows no significant effect for either the direct (β = 0.09, t = 0.94, *p* = 0.38, 95% CI = −0.13, 0.31) nor the indirect model (β = 0.00, SE = 0.16, 95% CI = −0.14, 0.32).

This model was also analysed using SDQ-P_Emotional problems as mediators and no significant effects (i.e., direct and indirect) emerged for either parents.

## 4. Discussion

According to the most recent epidemiological data in Italy, the onset of pediatric diabetes mostly occurs between 9 and 11 years of age [2,46]. In the context of pediatric diabetes, parents might be at greater risk of perceiving an intense level of parenting stress, due to their key role in the management of their child’s diabetes [12]. Most of the studies have focused on mothers’ parenting stress related to the children’s chronic illness, considering them as the primary caregivers involved in administering the insulin and the strict diet regimen [23,29]. As far as we know, very few studies have mentioned the absence of data regarding fathers as a limitation of the study. However, empirical evidence indicates that fathers’ psychosocial symptoms and parenting stress might influence the psychosocial functioning of children with T1DM more than comparative maternal variables [31]. Due to this evidence from the literature, the main aim of our paper was to assess both parents of school-aged children with T1DM, exploring differences between mothers and fathers on perceived parenting stress and perception of their child’s psychosocial symptoms.

Concerning the first hypothesis, mothers and fathers scored differently on their perception of internalizing symptoms in children with TDM1. More specifically, mothers reported higher levels compared to fathers. Notwithstanding the small sample size, it seemed that mothers were more preoccupied with the internal suffering of their children with T1DM who typically showed feelings of sadness and anxiety. The literature on the multiple-informant approach suggested that mothers are prone to detect higher levels of internalized symptoms in children compared to fathers [21]. This data could also find support in the fact that mothers might be more involved in medical diabetes management and in taking care of the mental state of their children. These data are partially in line with the literature, indicating that mothers reported children with TDM1 as more problematic than fathers, both in the internalizing and externalizing domains [18]. As regards parenting stress, mothers and fathers seemed to report comparable stress in parenting a child with diabetes, to the extent that both parents bear the affective burden of being a parent in the same way. A few studies compared mothers and fathers of school-aged children with T1DM regarding parenting stress, however, none of them discussed the results. The authors found that mothers reported higher levels of parenting stress compared to fathers, but in this case, the authors used a unique sample of children and adolescents with diabetes and asthma [29]. Other studies comparing mothers and fathers address a specific construct called *pediatric parenting stress*, designed around parents who care for a child with a chronic illness. While speculative, the results of the current study might be a function of the fact that parenting stress did not specifically address the fatigues of being a parent of a child with chronic illness, and so the possible greater involvement of the mother in the diabetes management did not affect the data. Future studies need to be carried out to provide more in-depth analyses on the differences and similarities between the two constructs and to evaluate the different implications in mothers’ and fathers’ involvement in their children’s diabetes.

According to the second hypothesis, parents’ perception of their children’s psychological symptoms is associated with the severity of their diabetes, measured by their scarce blood glycemic control [31]. Further explorations are needed to shed light on where this association could lead. Moreover, consistent with the above-mentioned hypothesis, a less than optimal glycemic control in children also seemed to be associated with greater parenting stress in both mothers and fathers. Despite the small sample size, the literature seems to confirm the same pattern, underlying that paternal parenting stress might be detrimental to children’s glycemic control, thereby in turn negatively affecting both parents’ involvement in their child-specific diabetes care [7]. The present study found a specific pattern in fathers, highlighting that scarce metabolic control in children was associated with an increase in paternal stress related to the perception of a dysfunctional interaction. Studies suggested that the evaluation of the interplay between psychosocial variables, parenting stress and medical outcome indicators might be useful to set up further clinical interventions [10,47]. For instance, targeting specific children with T1DM and both parents’ attitudes could help to ameliorate adherence to diabetes treatments [48] and, consequently, the child’s glycemic control.

With respect to the third and last hypothesis of the current study, poor metabolic control in children with T1DM when mediated by the fathers’ perception of related behavioral problems in their child seemed to further affect the fathers’ level of parenting stress. Very few studies performed mediational models in this area, and none of them ran the same models and took fathers specifically into account [9,10]. The findings of the present study are partially consistent with Limbers and colleagues (2018) data, suggesting that having children with diabetes affected the fathers’ level of parenting stress, when they recognized their children as displaying even more problematic behaviours, such as hyperactivity and distractibility. Our findings highlighted that scarce metabolic control, mediated by the fathers’ perception of their children’s behavioural problems, seemed to influence a specific aspect of parenting stress related to the dyad’s dysfunctional interaction. Some authors suggested that in diabetes management, the quality of the parent–child relationship and their communication is a crucial factor in the regulation of the blood glucose indexes [10]. However, this finding should be addressed and investigated in greater depth in future research. Overall the findings of the present paper indicate that it ought to be of interest to see how changes in a child’s HbA1c levels can affect parents’ perception of their child’s psychosocial functioning and also of their parenting stress.

### Limitations and Future Implications

The current study provides useful evidence in the research and clinical practice related to the interaction between parents and their children with T1DM. However, it is not without limitations, which can be addressed in future research. First, findings are based on small sample size and thus results cannot be generalized. Nevertheless, the study was not conducted in a controlled environment, thereby providing an ecological perspective of the parents’ perception of their child’s chronic disease and several studies with physiological measurements using a small sample size [49]. In addition, a non-parametric approach was performed in order to handle the small sample size.

This study used a cross-sectional design; nevertheless, it has been useful to identify relevant variables, which could be used for future longitudinal studies. The principal caregiver in the medical care of their children was not investigated, thus not permitting an in-depth understanding of certain data, which should be further examined. Besides, except for HbA1c, neither diabetes complications nor any other medical index levels were considered. The current study is, however, one of the few that took into account fathers and their perception of their child’s chronic disease. Indeed, parents’ perceptions, as well as their parenting stress, are relevant variables for the identification, in the clinical practice and how-to guide, and modulate their parenting behaviour in the interaction with their child. In this context, for instance, it could be useful to develop focus groups where parents can discuss their perceptions of their child’s diabetes with other parents. Such focus groups might also support them in parenting a child with T1DM. Moreover, it would be ideal for providers to investigate parental sources of stress and assess parental perceptions about the personal and family impact of illness, at the time of the diagnosis, during visiting hours and during educational weekends or annual summer-camps. Some psychological interventions should specifically focus on reducing mothers’ and fathers’ psychological distress through a decrease in the perception of difficulties in managing the child. In the Verona Paediatric Diabetes and Metabolic Disorders Unit, where the present study has been carried out, fathers traditionally attend scheduled psychological interventions less frequently during routine visits or annual summer-camps. So, providers need to encourage them to seek more frequent support from professionals, and they need to receive more adequate psychosocial counselling. Therefore, the present study attempts to identify important variables (i.e., parents’ perception of internalizing and externalizing problems in the child) to develop more accurate future interventions in the clinical practice for parents. More specifically, in the medical field, the improvement of the glycaemic control level is partially associated with progress in medical treatment, and at the same time, optimal psychological outcomes seem to be influenced by the family’s management of the psychosocial functioning of the child with diabetes.

## 5. Conclusions

The present study supports that mothers might be more involved than fathers in medical diabetes management, and in taking care of the mental state of their children, especially regarding the perception of a child with internalizing problems. These findings suggest that mothers reported children with T1DM as being more problematic compared to the perception of the fathers, with regards to the child’s internalizing and externalizing problems. Mothers and fathers reported comparable stress in parenting a child with diabetes, to the extent that both parents bear the affective burden of being a parent in the same way. Therefore, in both parents, parenting stress might be detrimental to children’s glycemic control, thereby in turn negatively affecting both parents’ involvement in their child-specific diabetes care. This study also sheds light on the fathers’ variables as regards their perception of managing their child with T1DM. Indeed, the father’s perception is crucial in the management of his child’s diabetes: scarce metabolic control, mediated by the fathers’ perception of their children’s behavioural problems seemed to have a role in a specific aspect of parenting stress related to the dyad’s dysfunctional interaction. Therefore, the current study provides useful evidence for clinical settings, suggesting that the interesting interplay between parenting stress, perception of children’s symptoms and glucometabolic control should be taken into consideration.

## Figures and Tables

**Table 1:** Wilcoxon test for non-independent sample—Perception of Parental Couples.

Paired *T*-Test	Mother Mean (SD)	Father Mean (SD)	Mean Difference (SD)	Z	Sig. (2 Tailed)
PSI_Total Stress Scale	71.08 (13.58)	64.75 (14.57)	6.33 (14.84)	−1.38	0.17
PSI_Parental Distress	26.83 (6.28)	22.42 (4.87)	4.42 (7.95)	−1.74	0.08
PSI_Parent–Child Dysfunctional Interaction	19.17 (4.45)	18.00 (3.93)	1.17 (5.47)	−.64	0.53
PSI_Difficult Child	25.08 (7.27)	24.50 (7.71)	0.58 (4.52)	−1.13	0.89
SDQ_Internalizing Symptoms	3.42 (3.43)	2.83 (2.79)	0.58 (0.90)	−1.93	0.04
SDQ_Externalizing Symptoms	5.67 (3.17)	5.33 (2.71)	0.33 (2.02)	−1.20	0.23
SDQ_Total Score	9.08 (6.36)	8.17 (5.31)	0.92 (2.19)		0.13
SDQ_Emotional Symptoms	2.17 (2.13)	1.92 (1.78)	0.25 (0.87)	−1.00	0.34
SDQ_Conduct Problems	2.08 (1.78)	2.00 (1.78)	0.08 (0.80)	−0.38	0.71

Note. *p* < 0.05, N = 12 couples, SDQ-P = Strengths and Difficulties Questionnaire–Parents, PSI-4-SF = Parenting Stress Index-4–Short Form.

**Table 2:** Partial Correlations between maternal perception of parenting stress (PSI-SF), maternal perception of children’s psychosocial functioning (SDQ-P), and HbA1c (mg/dL) of the child.

Mothers	HbA1c (mg/dL)	SDQ-P_Total Score	SDQ-P_ Internalizing	SDQ-P_ Externalizing	SDQ-P_Emotional Symptoms	SDQ-P_Conduct Problems	PSI_Total Stress	PSI_Parental Distress	PSI_Parent–Child Dysfunctional Interaction
SDQ-P_Total Score	0.85 ***	1.00							
SDQ-P_Internalizing	0.82 ***	**0.98** ***	1.00						
SDQ-P_Externalizing	0.85 ***	**0.98** ***	0.92 ***	1.00					
SDQ-P_Emotional Symptoms	0.84 ***	**0.78** **	0.80	0.73	1.00				
SDQ-P_Conduct Problems	0.93 ***	**0.89** ***	**0.83** ***	**0.92** ***	**0.84** ***	1.00			
PSI_Total Stress	0.47	0.48	0.56	0.38	0.53	0.49	1.00		
PSI_Parental Distress	−0.21	−0.14	−0.04	−0.24	0.00	−0.10	**0.65** **	1.00	
PSI_Parent–Child Dysfunctional Interaction	**0.68** **	**0.74** **	**0.83** ***	0.61	0.68 *	0.62	**0.82** ***	0.24	1.00
PSI_Difficult Child	0.69 **	0.60	0.60	0.57	0.61	**0.75** **	**0.85** ***	0.22	0.72 *

Note. All values in bold are significant at *p* < 0.05, low (*d* = 0.2) *, medium (*d* = 0.5) **, high (*d* = 0.8) ***, *n* = 12 couples, HbA1c = Hemoglobin A1C, SDQ-P = Strengths and Difficulties Questionnaire–Parents, PSI-4-SF = Parenting Stress Index–Short Form.

**Table 3:** Partial Correlations between paternal perception of parenting stress (PSI-4-SF), paternal perception of children’s psychosocial functioning (SDQ-P), and HbA1c (mg/dL) of the child.

Fathers	HbA1c (mg/dL)	SDQ-P_Total Score	SDQ-P_ Internalizing	SDQ-P_ Externalizing	SDQ-P_Emotional Symptoms	SDQ-P_Conduct Problems	PSI_Total Stress	PSI_Parental Distress	PSI_Parent–Child Dysfunctional Interaction
SDQ-P_Total Score	**0.78** **	1.00							
SDQ-P_Internalizing	**0.70** **	**0.98** ***	1.00						
SDQ-P_Externalizing	**0.84** ***	**0.98** ***	**0.93** ***	1.00					
SDQ-P_Emotional Symptoms	**0.73** **	**0.87** ***	**0.84** ***	**0.78** **	1.00				
SDQ-P_Conduct Problems	**0.90** ***	**0.81** ***	**0.75** **	**0.84** ***	**0.73** **	1.00			
PSI_Total Stress	**0.88** ***	**0.65** **	0.61	**0.67** **	0.60	**0.79** **	1.00		
PSI_Parental Distress	**0.72** **	0.55	0.48	0.60	0.37	**0.73** **	**0.85** ***	1.00	
PSI_Parent–Child Dysfunctional Interaction	0.58	0.21	0.13	0.29	0.21 *	0.27	**0.66** **	0.35	1.00
PSI_Difficult Child	**0.86** ***	**0.73** **	**0.72** **	**0.71** **	**0.74** **	**0.85** ***	**0.95** ***	**0.75** **	0.47 *

Note. All values in bold are significant at *p* < 0.05, low (*d* = 0.2) *, medium (*d* = 0.5) **, high (*d* = 0.8) ***, *n* = 12 couples, HbA1c = Hemoglobin A1C, SDQ-P = Strengths and Difficulties Questionnaire–Parents, PSI-4-SF = Parenting Stress Index–Short Form.
